# Microbiological and biochemical properties of soil polluted with a mixture of spiroxamine, tebuconazole, and triadimenol under the cultivation of *Triticum aestivum* L.

**DOI:** 10.1007/s10661-019-7539-4

**Published:** 2019-06-06

**Authors:** Małgorzata Baćmaga, Jan Kucharski, Jadwiga Wyszkowska

**Affiliations:** Department of Microbiology, University of Warmia and Mazury in Olsztyn, Plac Łódzki 3, 10-727 Olsztyn, Poland

**Keywords:** Fungicides, Soil environment, Microorganisms, Biodiversity, Enzymes, *Triticum aestivum* L

## Abstract

Pesticide contamination is one of the most serious threats for agricultural soils. Excessive pesticide levels in soil can exert negative effects on soil-dwelling organisms by decreasing their bioavailability and, consequently, lowering soil quality. This study aimed to evaluate the effect of a mixture of spiroxamine, tebuconazole, and triadimenol (S + Te + Tr) on the biological activity of soil determined based on the proliferation of microorganisms and their diversity, enzymatic activity of soil, and resistance of *Triticum aestivum* L. A pot experiment was performed on sandy loam (pH 7.0) treated with four doses of the tested active ingredients: 0.000, 0.092, 2.76, 13.80, and 27.60 mg kg^−1^. Soil without the fungicide served as the control sample. The tested fungicide induced changes in the biological activity of soil. When administered to the soil in the highest dose (27.60 mg kg^−1^ DM of soil), it inhibited its biological activity. It significantly inhibited the proliferation of organotrophs, actinomycetes, and fungi, but still the most susceptible to its effects turned out to be fungi. Fungicide dose of 27.60 mg kg^−1^ decreased counts of organotrophic bacteria, actinomycetes, and fungi by on average 0.009 log, 0.100 log, and 0.282 log, respectively, compared to the control sample. Administration of the S + Te + Tr mixture to soil decreased also values of colony development index (CD) determined for all tested groups of microorganisms. Values of the ecophysiological diversity index (EP) decreased in the case of organotrophs and actimomycetes and increased in the case of fungi. The S + Te + Tr mixture inhibited activities of dehydrogenases, urease, and acid phosphatase. Significant changes were also reported in the growth of spring wheat. The resistance index (RS) calculated based on plant yield demonstrated spring wheat to be very susceptible to the tested preparation administered to soil in doses of 13.80 and 27.60 mg kg^−1^.

## Introduction

Pesticides improve productivity and the quality of agricultural crops, which is why they are widely used around the world. From the economic point of view, pesticides are an effective tool for fighting pests in most agricultural sectors (Stenrød et al. 2013); yet, prolonged and improper use of those plant protection products can exert negative effects on the natural environment (Yang et al. 2012). When applied frequently during the growing season, pesticides can produce results that are contrary to their intended purpose, namely unnatural selection of living organisms that are not targeted by those chemicals. Pesticides are often used without sufficient knowledge about the risks associated with agricultural chemicals (Carvalho 2017). According to Muñoz-Leoz et al. (2011), only 0.3% of the product is used to fight pests, whereas remaining 99.7% is released into the environment and poses a threat for beneficial organisms. The highest levels of pesticide accumulation are found in soil. Chemicals may be subject biological or physicochemical changes in the soil environment, and microbial decomposition is the primary mechanism responsible for the elimination of those compounds from soil. Microbial metabolism plays a very important function in the degradation of xenobiotics in soil. Microorganisms capable of decomposing pesticides use those chemicals as a source of nutrients. Microbial breakdown of pesticides is determined by soil conditions and the persistence of active ingredients (Álvarez-Martín et al. 2016; Wyszkowska et al. 2016).

Despite microbial transformation, pesticides can exert a negative effect on soil organisms, including microorganisms. This leads, among other things, to lowering soil productivity. Some microorganisms are highly sensitive to pesticides, whereas others develop pesticide resistance mechanisms. Microorganisms and soil enzymes rapidly respond to stresses and are reliable bioindicators of changes in soil ecosystems (Lombard et al. 2011; Lupwayi et al. 2010). The presence and retention of pesticides in soil can have adverse consequences for the soil environment (Mahapatra et al. 2017; Niewiadomska et al. 2018). In most cases, pesticides inhibit the activity of soil-dwelling microorganisms. Soil-dwelling microbes are the first organisms to come into contact with soil pollutants, and they can be used as biological indicators to evaluate the severity of contamination with man-made chemicals (Garcίa-Gil et al. 2013; Ju et al. 2016; Mohiuddin and Mohammed 2013). Soil enzymes participate in various soil processes, and they can be used as environmental biomarkers based on their responses to soil stresses (Bennicelli et al. 2009). Intracellular and extracellular enzymes can catalyze biochemical processes. They participate in the circulation of elements, mineralization of organic compounds, and transformation of pollutants (Kızılkaya et al. 2012; Kumar et al. 2017; Wu et al. 2017). Soil enzymes are significantly influenced by pesticides which can exert a stimulating effect when applied in small amounts and inhibit enzyme activity when used in high doses (Saha et al. 2015; Tao and Yang 2011).

Spiroxamine is a ketoamine group compound which inhibits sterol biosynthesis. It is used to control powdery mildew in cereals and vegetables. Spiroxamine has a half-life of 37 to 44 days in soil. The compound is decomposed into four metabolites: spiroxamine despropyl, spiroxamine desethyl, spiroxamine aminodiol-N-oxide, and spiroxamine acid (Sukul et al. 2010). Tebuconazole is a systemic fungicide from the triazole group. It is applied to control fungal diseases in crop plants, mainly cereals and maize. Tebuconazole inhibits the biosynthesis of sterols and disrupts the synthesis of fungal cell walls. The compound is characterized by high persistence and low or moderate mobility in soil (Ahemad and Khan 2012; Herrero-Hernández et al. 2011). Its half-life under aerobic conditions in soil is estimated at 49 to 610 days (Muñoz-Leoz et al. 2011). Triadimenol is a systemic fungicide from the triazole group. It is applied to control powdery mildew. Triadimenol has two chiral centers and one to two enantiomeric pairs. It occurs in four steroisomeric forms: (1R, 2S)-triadimenol, (1S, 2R)-triadimenol, (1R, 2R)-triadimenol, and (1S, 2S)-triadimenol (Dong et al. 2010).

The main objective of this study was to evaluate changes in the soil environment induced by a combination of three active substances (spiroxamine, tebuconazole, triadimenol) based on microbial abundance and biodiversity, enzyme activity levels, soil resistance, and resilience values. According to our best knowledge, there are no comprehensive assays for evaluating the combined impact of those compounds on the biological activity of soil. A knowledge of the toxic effects of the tested compounds on soil-dwelling microorganisms can be used to develop threshold doses of pesticides that do not have a detrimental impact on the soil environment.

## Materials and methods

### Soil

The brown soil formed from sandy clay was taken from the area of north-eastern part of Poland (53.71610°N, 20.41670°E), located in Central Europe. The granulometric composition of sandy loam, physicochemical parameters of soil, and determination methods are described in a work by Baćmaga et al. (2016).

### Fungicide

The fungicide used in the study was Falcon 460 EC, whose characteristics is given in the study by Baćmaga et al. (2016). PEC (predicted environmental concentrations) of the tested active substances are given in Table 1.

**Table 1:** The predicted environmental concentrations of active ingredients in soil, mg kg^−1^

Active ingredient dose (mg kg^−1^)	Predicted environmental concentrations of active ingredients in soil (days)	
	25	50
Spiroxamine		
0.050	0.025	0.013
1.500	0.750	0.375
7.500	3.750	1.875
15.00	7.500	3.750
Tebuconazole		
0.033	0.022	0.019
0.990	0.752	0.571
4.950	3.759	2.856
9.900	7.519	5.711
Triadimenol		
0.009	0.007	0.005
0.270	0.207	0.158
1.350	1.034	0.791
2.700	2.067	1.583

### Experimental design

A pot experiment was conducted in a greenhouse (four replications). Polyethylene pots were filled with 3-kg samples of sandy loam each. The soil was combined with the respective dose of the fungicide and mineral fertilizers. A mixture of S + Te + Tr was used to soil samples in doses: 0.000, 0.092, 2.76, 13.80, and 27.60 mg kg^−1^. Mineral fertilizers were applied in accordance with the requirements for spring wheat. The moisture content of the soil material was 50%. After 7 days, spring wheat (*Triticum aestivum* L.) cv. Torka was sown (12 plants per pot). At 25 and 50 days after sowing, soil samples were collected from pots with different doses of the spiroxamine, tebuconazole, and triadimenol mixture to create a pooled sample with the weight of 400 g, in order to perform enzymatic and microbiological analyses.

### Soil microbiological analysis

Soil materials were subjected to microbiological analyses to determine the number of actinomycetes, fungi, and organotrophic bacteria (three replications). A detailed methodology used to determine numbers of soil microorganisms was described by Kucharski et al. (2016). On the basis the number of microbial, the colony development (CD) index (Sarathchandra et al. 1997) and the ecophysiological diversity (EP) index of microorganisms (De Leij et al. 1993) were calculated. The number of colonies formed over specified time intervals (K_s_) was determined according to formula by Tomkiel et al. (2015).

### Soil enzymatic activity

The enzyme activities (acid phosphatase, alkaline phosphatase, catalase, dehydrogenases, and urease) were determined (on days 25 and 50) in the soil samples in three replications. The determine procedure was the same as in the study by Borowik et al. (2017).

### Resistance of spring wheat to soil contamination with a mixture of S + Te + Tr

On day 7, seeds of *Triticum aestivum* L. cv. Torka (25 pieces) were seeded per pot. After the germination of the seeds, 12 plants were left per pot. *Triticum aestivum* L. was collected on day 50 at the BBCH 52 heading stage. Aboveground plant parts were dried at 65 °C, dry matter yield was determined, and the results were used to calculate spring wheat’s resistance (RS) to the tested fungicide (Orwin and Wardle 2004).

### Statistical analysis

The results were analyzed statistically using the Statistica 12.0 software (Stat. Soft Inc. 2015) with the use of two-way ANOVA (at significance level of *p* = 0.05). Homogeneous groups were determined in Tukey’s range test. The abundance of soil-dwelling microorganisms was presented in a dendrogram with the use of cluster analysis (Ward’s method), and soil enzymatic activity was presented with the use of principal component analysis. The correlation coefficients between the fungicide dose and the analyzed parameters were calculated. The proportion of variance explained in microbial abundance and enzyme activity was calculated with the use of coefficient η^2^.

## Results and discussion

### Soil microorganisms

**Fig. 1:**
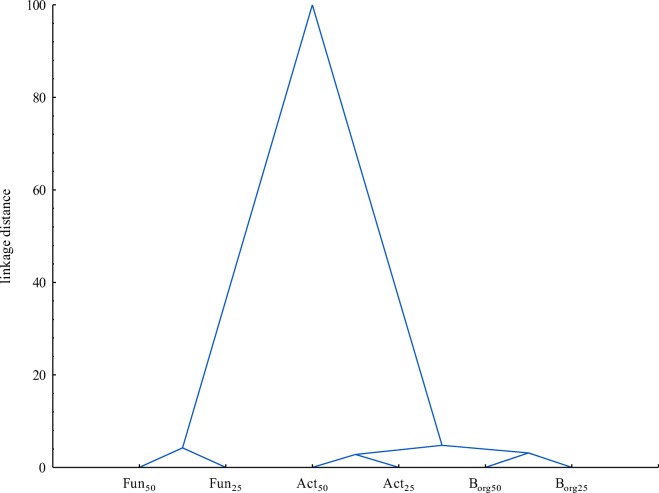
Similar responses of soil-dwelling microorganisms to contamination with the mixture of S + Te + Tr. Microorganisms: Borg–organotrophic bacteria, Act–actinomycetes, Fun–fungi. Term analysis: 25 and 50 days

The effect of the fungicide on the number of microorganisms was dependent on its dose and the time of retention in the soil. It has been shown that the mixture of S + Te + Tr dose influenced the numbers of organotrophic bacteria in 35.74%, actinomycetes in 33.71% and fungi in 51.07%. The date of analysis was a less influential factor in microbial proliferation. Fungi were most sensitive to the applied mixture of S + Te + Tr (Table 2), as revealed by the highest negative correlation coefficients between fungicide dose and fungal abundance (*r* = − 0.941 on day 25, *r* = − 0.947 on day 50). The fungicide dose of 27.60 mg kg^−1^ decreased fungal numbers by 0.264 log on day 25 and by 0.300 log on day 50 relative to the control sample. This response of fungi to S + Te + Tr mixture stems from the fact that it is used in plant protection against fungal pathogens. Hence, fungi proliferation in the soil environment was significantly inhibited (Baćmaga et al. 2016). Organotrophic bacteria and actinomycetes produced more varied responses to the tested preparation. Counts of organotrophic bacteria in the soil non-contaminated with S + Te + Tr mixture reached 9.946 log cfu kg^−1^ on day 25 and 9.903 log cfu kg^−1^on day 50 of the experiment. On day 25, fungicide doses between 2.760 and 27.60 mg kg^−1^ decreased the numbers of organotrophic bacteria by 0.113 to 0.212 log, respectively, but on day 50, they exerted a stimulating effect on bacterial proliferation (population number increase from 0.032 log to 0.149 log). The observed enhanced proliferation of organotrophic bacteria could be due to their capability for fungicide degradation to less toxic compounds and for simultaneous use of active substances of the tested preparation as sources of energy and nutrients (Bishnu et al. 2012). These microorganisms could be more tolerant to S + Te + Tr mixture and, by this means, could adapt to unfavorable conditions it caused (Xu et al. 2014). In addition, once the fungicide had been administered to the soil, organotrophic bacteria did not have to compete with fungi, and these circumstances afforded favorable conditions for their growth and development (Cycoń et al. 2010). The highest numbers of organotrophic bacteria (10.052 log cfu kg^−1^) were noted in pots with the fungicide dose recommended. When applied in higher doses, the fungicide inhibited the proliferation of actinomycetes. The number of actinomycetes in the control soil accounted for 9.706 log cfu kg^−1^ on day 25 and for 9.861 log cfu kg^−1^ on day 50. The highest dose (27.60 mg kg^−1^) reduced actinomycete numbers by 0.04 log on day 25 and by 0.160 log on day 50. The inhibition of actinomycete growth could be induced by the additive or synergistic effects of active substances of the preparation (Baćmaga et al. 2016; Tejada 2009). A decrease in their population could also be due to the formation of more toxic metabolites during degradation of active substances in the soil, which inhibited the development of microorganisms (Muñoz-Leoz et al. 2013). The adverse impact of the mixture of S + Te + Tr on soil-dwelling microorganisms could ensue from the synergistic effect of those active ingredients (Tejada 2009) or the formation of secondary metabolites having greater toxicity than the original compound (Bello et al. 2008). Microbial responses to the tested preparation were presented in a dendrogram with the use of cluster analysis (Fig. 1). Two homogeneous groups can be identified in the diagram. The first group is composed of actinomycetes and organotrophic which reacted in a similar manner to soil contamination with the tested fungicide. The second cluster was formed by fungi which were most sensitive to the analyzed product. Pesticides generally induce morphological changes in microorganisms and decrease their biological activity. Negative impacts of fungicides on the population of soil microorganisms were also reported by Baćmaga et al. (2018), Guo et al. (2015), Muñoz-Leoz et al. (2011), and Saha et al. (2016).

**Table 2:** Microbial number in soil contaminated with the mixture of S + Te + Tr, log cfu kg^−1^ DM soil

Dose S + Te + Tr mg kg^−1^	Organotrophic bacteria		Actinomycetes		Fungi	
	Term analysis, days					
	25	50	25	50	25	50
0.000	9.946^*abc*^	9.903^*bc*^	9.706^*a*^	9.861^*a*^	7.511^*cd*^	7.715^*abc*^
0.092	10.030^*a*^	10.052^*a*^	9.837^*a*^	9.733^*a*^	7.579^*cd*^	7.829^*a*^
2.760	9.833^*cd*^	10.027^*ab*^	9.771^*a*^	9.752^*a*^	7.431^*de*^	7.755^*bcd*^
13.80	9.737^*d*^	10.004^*ab*^	9.696^*a*^	9.741^*a*^	7.365^*de*^	7.511^*cd*^
27.60	9.734^*d*^	9.935^*abc*^	9.666^*a*^	9.701^*a*^	7.247^*e*^	7.415^*de*^
$\overline{x}$	9.856	9.984	9.735	9.758	7.427	7.645
*r*	− 0.814	− 0.320	− 0.716	− 0.624	− 0.941	− 0.947

$\overline{x}$ average, *r* coefficient of correlation. Identical letters in columns denote homogeneous groups within a given microbial group

According to Sułowicz and Piotrowska-Seget (2016), improper use of pesticides can provoke modification in the biodiversity and structure of soil-dwelling microorganisms. The CD index and EP index presented in Table 3 provide valuable information about the microbiological condition of soil. The lowest average values of the CD index were noted in actinomycetes on experimental days 25 and 50 (35.766 and 30.439, respectively), whereas fungi CD values were the highest on these dates (38.369 and 43.560, respectively). According to Sarathchandra et al. (1997), higher CD values point to a greater advantage of r-strategists (rapidly proliferating microorganisms which colonize less stable habitats) over K-strategists (slowly proliferating or dormant microorganisms). Baćmaga et al. (2015) observed that CD values of all tested microorganisms increased under the Alister Grande 190 OD preparation. In present research, exposure to high doses of spiroxamine, tebuconazole, and triadimenol decreased the CD index of fungi and increased the CD values of organotrophic bacteria and actinomycetes. The S + Te + Tr mixture inhibited the rate of soil microbiota proliferation and this caused changes in microbiota structure, i.e., a higher number of more slowly growing species than of fast-growing species. This change may result from disorders in the synthesis of the cell wall of microorganisms evoked by the fungicide that contributed to their reduced count and, consequently, to a shift in the structure of microbial communites (Cycoń and Piotrowska-Seget 2015). Somewhat different relationships were noted in EP values. On days 25 and 50, the lowest average EP values were noted in fungi (0.552 and 0.421, respectively), and the highest average EP values in organotrophic bacteria (0.792 and 0.686, respectively). These results proved that diversity of fungi increased, while that of organotrophic bacteria and actinomycetes decreased. This may be due to the fact that increased doses of the fungicide caused the appearance of new fungal species being more tolerant to the S + Te + Tr mixture. In addition, substances released from dead cells of microorganisms could have been used by fungi as nutrients (Cycoń and Piotrowska-Seget 2015).

**Table 3:** The colony development (CD) index and the ecophysiological diversity (EP) index in soil contaminated with the mixture of S + Te + Tr

Dose S + Te + Tr mg kg^−1^	Organotrophic bacteria		Actinomycetes		Fungi	
	Term analysis, days					
	25	50	25	50	25	50
Colony development (CD) index						
0.000	41.131^*ab*^	37.197^*b*^	36.373^*ab*^	27.142^*c*^	46.066^*ab*^	46.057^*ab*^
0.092	41543^*ab*^	35.323^*bc*^	39.169^*a*^	27.432^*c*^	37.208^*bc*^	46.576^*a*^
2.760	33.842^*bc*^	35.830^*bc*^	37.232^*ab*^	31.415^*b*^	37.018^*bc*^	45.077^*ab*^
13.80	32.687^*c*^	35.554^*bc*^	36.696^*ab*^	32.909^*b*^	35.611^*c*^	45.011^*ab*^
27.60	31.726^*c*^	45.191^*a*^	29.359^*bc*^	33.298^*b*^	35.940^*c*^	35.077^*c*^
$\overline{x}$	36.186	37.819	35.766	30.439	38.369	43.560
*r*	− 0.781	0.835	− 0.895	0.817	− 0.524	− 0.918
Ecophysiological diversity (EP) index						
0.000	0.736^*b*^	0.713^*b*^	0.651^*b*^	0.753^*a*^	0.428^*bcd*^	0.347^*d*^
0.092	0.747^*b*^	0.765^*ab*^	0.660^*b*^	0.790^*a*^	0.557^*abc*^	0.378^*cd*^
2.760	0.806^*ab*^	0.776^*ab*^	0.651^*b*^	0.767^*a*^	0.581^*abc*^	0.382^*bcd*^
13.80	0.876^*a*^	0.744^*b*^	0.659^*b*^	0.729^*a*^	0.621^*a*^	0.408^*bc*^
27.60	0.795^*ab*^	0.434^*c*^	0.667^*b*^	0.715^*a*^	0.573^*abc*^	0.589^*ab*^
$\overline{x}$	0.792	0.686	0.658	0.751	0.552	0.421
*r*	0.480	− 0.867	0.808	− 0.872	0.462	0.947

$\overline{x}$ – average, *r* coefficient of correlation. Identical letters in columns denote homogeneous groups within a given microbial group, separately for CD and EP

The microbial growth rate (Ks) over a given time period plays an important function in the maintenance of soil ecosystem homeostasis (Table 4). In the control treatment, the greatest modifications were found in the first 2 days of incubation. Microbial growth rates were determined at 46.897% for organotrophic bacteria, 44.142% for actinomycetes, and 82.960% for fungi on day 25, and at 49.099%, 28.652%, and 82.766%, respectively, on day 50. Different results were noted in treatments contaminated with the mixture of S + Te + Tr. On day 25 and day 50, organotrophic bacteria and fungi were the most abundant microorganisms. On day 25, the highest proliferation of actinomycetes was noted over a period of 2 days, and on day 50 over a period of 4 days. These results suggest that the most intensive proliferation of microorganisms occurred in the first 4 days of the culture, which confirms that changes in the soil environment could determine the predominance of r-strategists. Metabolites formed during fungicide degradation could have been used as substrates for the growth of r-strategists, i.e., microorganisms capable of proliferating in the nutrient-rich environment (Cycoń et al. 2010). In a study of carfentrazone-ethyl, Tomkiel et al. (2015) demonstrated that microorganisms proliferate most rapidly in the first 4 days of incubation.

**Table 4:** The combined effect of S + Te + Tr on the number of microbial colonies (%) formed over specified time intervals (Ks)

Dose S + Te + Tr mg kg^−1^	Term analysis, days									
	25					50				
	Days of culture									
	1–2	3–4	5–6	7–8	9–10	1–2	3–4	5–6	7–8	9–10
Organotrophic bacteria										
0.000	46.897	31.837	14.690	6.576	0.000	49.099	23.385	18.612	7.093	1.811
0.092	54.791	25.424	11.250	8.535	0.000	35.402	29.756	18.942	14.395	1.506
2.760	55.668	27.709	10.393	6.230	0.000	30.114	34.301	20.639	13.863	1.084
13.80	43.223	39.376	10.060	7.341	0.000	29.925	35.710	19.115	13.829	1.421
27.60	44.619	36.760	11.477	7.144	0.000	32.414	31.684	22.677	12.888	0.337
$\overline{x}$	49.040	32.221	11.574	7.165	0.000	35.391	30.967	19.997	12.414	1.232
*r*	− 0.662	0.752	− 0.307	− 0.015	–	− 0.453	0.410	0.789	0.255	− 0.837
Actinomycetes										
0.000	44.142	34.348	15.177	3.575	2.757	28.652	16.404	15.465	36.384	3.096
0.092	37.494	48.331	8.188	5.987	0.000	23.429	27.845	29.222	16.620	2.884
2.760	36.318	47.040	9.306	6.771	0.565	23.546	31.136	25.954	16.223	3.141
13.80	43.540	34.476	16.742	4.677	0.565	25.545	35.438	24.344	14.018	0.654
27.60	39.750	29.465	22.363	7.858	0.565	28.431	39.615	21.859	9.441	0.654
$\overline{x}$	40.249	38.732	14.355	5.774	0.890	25.921	30.088	23.369	18.537	2.086
*r*	0.123	− 0.723	0.868	0.561	− 0.283	0.474	0.805	− 0.083	− 0.647	− 0.901
Fungi										
0.000	82.960	12.855	4.185	0.000	0.000	82.766	11.228	6.006	0.000	0.000
0.092	63.343	19.345	15.460	1.852	0.000	77.961	12.558	8.626	0.855	0.000
2.760	55.956	20.566	21.626	1.852	0.000	75.160	13.959	10.027	0.855	0.000
13.80	61.049	15.937	23.015	0.000	0.000	62.339	23.645	14.016	0.000	0.000
27.60	57.924	15.937	26.140	0.000	0.000	39.006	35.867	18.460	6.667	0.000
$\overline{x}$	64.246	16.928	18.085	0.741	0.000	67.446	19.451	11.427	1.675	0.000
*r*	− 0.468	− 0.210	0.724	− 0.569	–	− 0.993	0.999	0.970	0.835	–

$\overline{x}$ – average, *r* coefficient of correlation

### Soil enzymatic activity

**Fig. 2:**
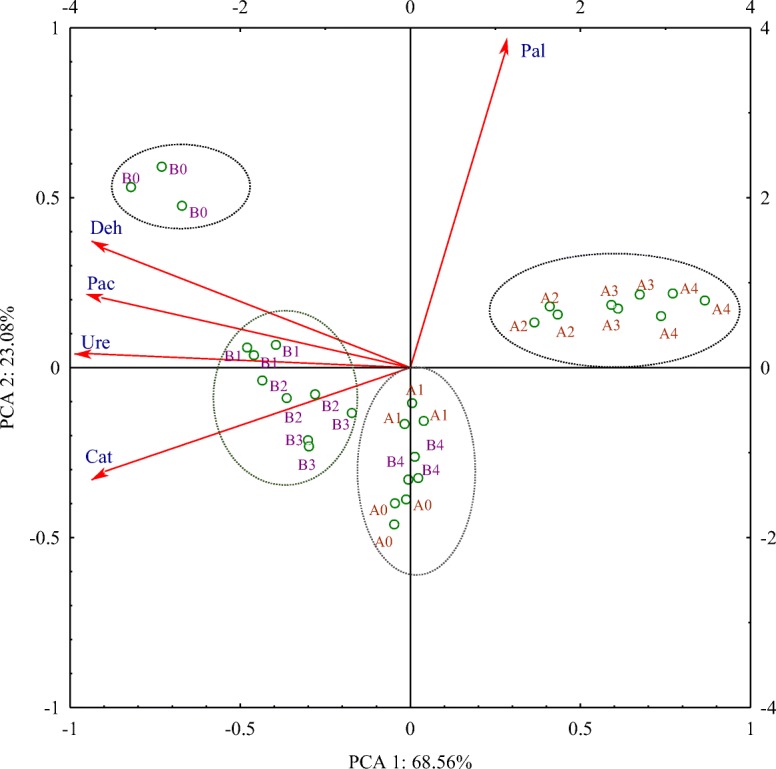
Changes in enzyme activity in response to soil contamination with the mixture of S + Te + Tr, presented by the PCA method. Enzymes: Deh–dehydrogenases, Cat–catalase, Ure–urease, Pac–acid phosphatase, Pal–alkaline phosphatase. Combined dose of active ingredients in 1 kg DM soil: 0–0 mg (control), 1–0.092 mg, 2–2.760 mg, 3–13.800 mg, 4–27.600 mg. Term analysis: A–25 days, B–50 days

The calculated proportion of variance explained η^2^ indicates that the dose of mixture of S + Te + Tr had the greatest impact on the activity of acid phosphatase (60.10%), whereas time was most likely to determine the activity of urease (81.01%). The tested fungicide inhibited the activity of all soil enzymes, excluding alkaline phosphatase (activity in the 25 days was stimulated). The negative correlation coefficients between fungicide dose and enzyme activity (Table 5) point to the adverse effects of the mixture of S + Te + Tr. Regardless of the fungicide’s retention time, the dose of 0.092 mg kg^−1^ induced only a minor reduction in enzyme activity. The highest changes in soil enzymatic activity were observed under exposure to the highest dose (27.60 mg kg^−1^). The analyzed enzymes can be placed in the order from the most sensitive to the most resistant (based on their average sensitivity to the 27.60-mg kg^−1^ dose of the tested fungicide): urease (decrease by 36.47%) > dehydrogenases (decrease by 26.57%) > acid phosphatase (decrease by 14.38%) > catalase (decrease by 11.89%) > alkaline phosphatase (decrease by 2.86%). The S + Te + Tr mixture caused the highest inhibition of urease activity. In the soil environment, this enzyme is strongly associated with organic matter and silty minerals and therefore exhibits higher stability and durability. A significant decrease in its activity may be explained by the fact that the soil used in the experiment could contain an insufficient amount of organic nitrogen compounds indispensable for the proliferation of microorganisms taking part in urea hydrolysis (Rahmansyah et al. 2009). It may also be speculated that excess doses of the fungicide triggered changes in the population of microorganisms, which reduced urease production and its release to the soil environment. For this reason, this enzyme became the most susceptible to stress conditions developed in the soil contaminated with S + Te + Tr mixture (Moreno et al. 2007). These results are consistent with findings reported by Muñoz-Leoz et al. (2011) and Baćmaga et al. (2016). Dehydrogenases and catalase are also reliable indicators of soil biological activity. Those enzymes belong to the class of oxidoreductases which are present in the respiratory chain of soil microorganisms (Bello et al. 2013). In this study, oxidoreductases responded negatively to the combined influence of active substances. The suppressed activity of dehydrogenases in the soil exposed to the S + Te + Tr mixture could be due to the damage of cells of microorganisms susceptible to the fungicide. Dehydrogenases are representatives of intracellular enzymes whose activity is associated with live cells of microorganisms. For this reason, enzymes released during cell lysis are not accumulated in the soil but undergo rapid denaturation and degradation (Saha et al. 2016). The sensitivity of dehydrogenases to fungicides was confirmed by Chen et al. (2001) in a study of benomyl and captan and by Jastrzębska and Kucharski (2007) who tested two preparations of fungicides: Unix 75 WG and Swing Top 183 SC. In the present study, catalase activity was inhibited and acid phosphatase was highly sensitive to the tested fungicide. In turn, alkaline phosphatase exhibited various responses, i.e., the S + Te + Tr mixture stimulated its activity on day 25 and inhibited it on day 50. Phosphatases are extracellular enzymes bound by soil colloids which protect them. In addition, excess doses of the tested preparation could trigger changes in conditions occurring in the soil, thus contributing to the suppressed activity of these enzymes (Abbas et al. 2015). Decreased activity of alkaline phosphatase at day 50 could be due to a decrease caused by the fungicide in the proliferation of fungi which are the main producers of this enzyme (Cycoń et al. 2010). Such a dependency was also observed in the study conducted by Baćmaga et al. (2018) with chlorothalonil. The results of PCA (Fig. 2) confirmed that spiroxamine, tebuconazole, and triadimenol exerted a negative effect on soil enzymes. The variations in the activity the analyzed enzymes were explained in 68.56% by the PCA 1 and in 23.08% by the PCA 2. The PCA 1 was negatively correlated with dehydrogenases, catalase, urease, and acid phosphatase, and the PCA 2 with alkaline phosphatase. The arrangement of cases in the PCA scatterplot indicates that the highest fungicide dose of 27.60 mg kg^−1^ had the most contributed to the changes in soil enzymatic activity. Enzyme activity was also influenced by the retention time of mixture of S + Te + Tr in soil. The inhibitory influence of the tested fungicide on enzyme activity you can be attributed to the presence of residual active ingredients in soil. The above is confirmed by the PEC values of active ingredients in Table 1, indicating that the greatest changes in enzymatic activity could be induced by tebuconazole. The inhibitory effect of the S + Te + Tr mixture on the enzymatic activity of soil may be associated with the inhibition of microorganism proliferation caused by disturbed metabolic processes in their cells, and even by their death. This adverse effect of the fungicide on soil microorganisms could indirectly contribute to reduced synthesis of enzymes participating in biogeochemical cycles (Saha et al. 2016). The inactivating effects of fungicides on activities of soil enzymes were earlier reported by Raju and Venkateswarlu (2013), Sułowicz and Piotrowska-Seget (2016), and Wang et al. (2016). In addition, these authors confirmed a strong correlation between the activity of soil enzymes and the proliferation of soil microorganisms.

**Table 5:** Enzyme activity in soil contaminated with the mixture of S + Te + Tr, 1 kg DM h^−1^

Dose S + Te + Tr mg kg^−1^	Dehydrogenases (μMol TPF)		Catalase (Mol 0_2_)		Urease (mMol N-NH_4_)		Acid phosphatase (mMol PNP)		Alkaline phosphatase (mMol PNP)	
	Term analysis, days									
	25	50	25	50	25	50	25	50	25	50
0.000	6.137^*fg*^	13.028^*a*^	0.350^*a*^	0.348^*a*^	0.060^*e*^	0.110^*a*^	0.964^*b*^	1.088^*a*^	1.344^*e*^	1.946^*a*^
0.092	7.470^*de*^	11.001^*b*^	0.334^*ab*^	0.345^*ab*^	0.055^*f*^	0.106^*a*^	0.982^*b*^	0.977^*b*^	1.507^*d*^	1.609^*c*^
2.760	6.914^*ef*^	10.095^*bc*^	0.312^*cd*^	0.341^*ab*^	0.045^*g*^	0.099^*b*^	0.908^*c*^	0.977^*b*^	1.817^*b*^	1.500^*d*^
13.80	5.931^*f*^	9.394^*c*^	0.301^*de*^	0.340^*ab*^	0.030^*h*^	0.085^*c*^	0.892^*c*^	0.970^*b*^	1.867^*ab*^	1.406^*e*^
27.60	5.710^*f*^	8.363^*d*^	0.289^*e*^	0.326^*bc*^	0.030^*h*^	0.078^*d*^	0.847^*d*^	0.910^*c*^	1.867^*ab*^	1.329^*e*^
$\overline{x}$	6.432	10.376	0.317	0.340	0.044	0.096	0.919	0.984	1.680	1.558
*r*	− 0.724	− 0.828	− 0.861	− 0.951	− 0.871	− 0.953	− 0.905	− 0.741	0.705	− 0.747

$\overline{x}$ – average, *r* coefficient of correlation. Identical letters in columns denote homogeneous groups within a given enzyme group

### Resistance of *Triticum aestivum* L. to soil contamination with the mixture of S + Te + Tr

**Fig. 3:**
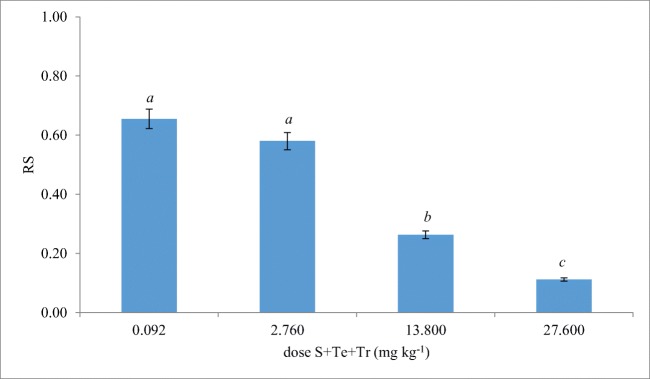
Resistance (RS) of spring wheat to soil contamination with the mixture of S + Te + Tr. Identical letters denote homogeneous groups. Error bars represent standard deviation (*n* = 3)

In our study, the RS (resistance index) values of spring wheat exposed to the S + Te + Tr mixture indicate that it hindered the increase of the experimental crops (Fig. 3). Spring wheat was most sensitive to fungicide doses of 13.800 mg kg^−1^ and 27.600 mg kg^−1^, and, RS values were determined at 0.263 and 0.112, respectively. The increased sensitivity of spring wheat to the excess doses of the tested fungicide could be due to disorders in plant metabolism. The fungicide could lead to damages of, i.e., chloroplasts, which inhibited the photosynthesis process and retarded nutrients migration in spring wheat plants. As a consequence, these changes led to decreased plant yield and plant resistance to the fungicide (Yuan et al. 2013). In addition, test plant growth inhibition could result from the synergistic effect of the S + Te + Tr mixture, which could exhibit higher toxicity compared to a single active substance. The negative effect of fungicides administered in a dose 100 times exceeding their optimal dose on crop growth was earlier confirmed by Baćmaga et al. (2018) and by Jastrzębska and Kucharski (2007). In the first mentioned study investigating the effect of chlorothalonil on spring wheat growth, authors observed that resistance of the test plant decreased along with increasing soil contamination with the fungicide. In the second mentioned research, the yield of spring barley decreased upon soil contamination with such fungicides as: cipronidil and a mixture of dimoxystrobin and epoxiconazole.

## Conclusions

Overuse of chemical pesticides can contaminate farmland, making it unsuitable for agricultural production. Comprehensive analyses are required to assess the influence of pesticides on soil quality and monitor the resulting changes in the soil ecosystem. Analyses of soil biological parameters deliver reliable results. Our findings indicate that excessive doses of the S + Te + Tr mixture adversely affect to the soil ecosystems. The tested compounds exerted a negative effect on the number and biodiversity of soil-dwelling microorganisms, enzyme activity, and spring wheat yield. The predicted environmental concentrations of active ingredients indicate that the tested product should be applied with great caution. Our findings provide valuable data for monitoring soil environments exposed to pesticides.

## References

[CR1] Abbas Z, Akmal M, Khan KS, Hassan FU (2015). Impacts of long-term application of buctril super (bromoxynil) herbicide on microbial population, enzymes activity, nitrate nitrogen, Olsen-P and total organic carbon in soil. Archives of Agronomy and Soil Science.

[CR2] Ahemad M, Khan MS (2012). Productivity of greengram in tebuconazole-stressed soil, by using a tolerant and plant growth-promoting *Bradyrhizobium* sp. MRM6 strain. Acta Physiologiale Plantarum.

[CR3] Álvarez-Martín A, Hilton SL, Bending GD, Rodríguez-Cruz MS, Sánchez-Martín MJ (2016). Changes in activity and structure of the soil microbial community after application of azoxystrobin or pirimicarb and an organic amendment to an agricultural soil. Applied Soil Ecology.

[CR4] Baćmaga M, Borowik A, Kucharski J, Tomkiel M, Wyszkowska J (2015). Microbial and enzymatic activity of soil contaminated with a mixture of diflufenican+mesosulfuron-methyl+iodosulfuron-methyl-sodium. Environmental Science and Pollution Research.

[CR5] Baćmaga M, Wyszkowska J, Kucharski J (2016). The effect of the falcon 460 EC fungicide on soil microbial communities, enzyme activities, and plant growth. Ecotoxicology.

[CR6] Baćmaga M, Wyszkowska J, Kucharski J (2018). The influence of chlorothalonil on the activity of soil microorganisms and enzymes. Ecotoxicology.

[CR7] Bello D, Trasar-Cepeda C, Leiro’s MC, Gil-Sotres F (2008). Evaluation of various tests for the diagnosis of soil contamination by 2,4,5-trichlorophenol (2,4,5-TCP). Environmental Pollution.

[CR8] Bello D, Trasar-Cepeda C, Leirós MC, Gil-Sotres F (2013). Modification of enzymatic activity in soils of contrasting pH contaminated with 2,4-dichlorophenol and 2,4,5-trichlorophenol. Soil Biology and Biochemistry.

[CR9] Bennicelli RP, Szafranek-Nakonieczna A, Woliñska A, Stępniewska Z, Bogudzińska M (2009). Influence of pesticide (glyphosate) on dehydrogenase activity, pH, eh and gases production in soil (laboratory conditions). International Agrophysics.

[CR10] Bishnu A, Chakraborty A, Chakrabarti K, Saha T (2012). Ethion degradation and its correlation with microbial and biochemical parameters of tea soils. Biology and Fertility of Soils.

[CR11] Borowik A, Wyszkowska J, Wyszkowski M (2017). Resistance of aerobic microorganisms and soil enzyme response to soil contamination with Ekodiesel ultra fuel. Environmental Science and Pollution Research.

[CR12] Carvalho FP (2017). Pesticides, environment, and food safety. Food and Energy Security.

[CR13] Chen SK, Edwards CA, Subler S (2001). Effects of the fungicides benomyl, captan and chlorothalonil on soil microbial activity and nitrogen dynamics in laboratory incubations. Soil Biology and Biochemistry.

[CR14] Cycoń M, Piotrowska-Seget Z (2015). Biochemical and microbial soil functioning after application of the insecticide imidacloprid. Journal of Environmental Sciences.

[CR15] Cycoń M, Piotrowska-Seget Z, Kozdrój J (2010). Responses of indigenous microorganisms to a fungicidal mixture of mancozeb and dimethomorph added to sandy soils. International Biodeterioration & Biodegradation.

[CR16] De Leij FAAM, Whipps JM, Lynch JM (1993). The use of colony development for the characterization of bacterial communities in soil and on roots. Microbial Ecology.

[CR17] Dong F, Liu X, Zheng Y, Cao Q, Li C (2010). Stereoselective degradation of fungicide triadimenol in cucumber plants. Chirality.

[CR18] Garcίa-Gil JC, Kobza J, Soler-Rovira P, Javoreková S (2013). Soil microbial and enzyme activities response to pollution near an aluminium smelter. Clean: Soil, Air, Water.

[CR19] Guo P, Zhu L, Wang J, Wang J, Xie H, Lv D (2015). Enzymatic activities and microbial biomass in black soil as affected by azoxystrobin. Environmental Earth Sciences.

[CR20] Herrero-Hernández E, Andrades MS, Marίn-Benito JM, Sánchez-Martίn MJ, Rodrίguez-Cruz MS (2011). Field-scale dissipation of tebuconazole in a vineyard soil amended with spent mushroom substrate and its potential environmental impact. Ecotoxicology and Environmental Safety.

[CR21] Jastrzębska E, Kucharski J (2007). Dehydrogenases, urease and phosphatases activities of soil contaminated with fungicides. Plant, Soil and Environment.

[CR22] Ju C, Xu J, Wu X, Dong F, Liu X, Zheng Y (2016). Effects of myclobutanil on soil microbial biomass, respiration, and soil nitrogen transformations. Environmental Pollution.

[CR23] Kızılkaya R, Akça İ, Aşkın T, Yılmaz R, Olekhov V, Samofalova I, Mudrykh N (2012). Effect of soil contamination with azadirachtin on dehydrogenase and catalase activity of soil. Eurasian Journal of Soil Science.

[CR24] Kucharski J, Tomkiel M, Baćmaga M, Borowik A, Wyszkowska J (2016). Enzyme activity and microorganisms diversity in soil contaminated with the boreal 58 WG herbicide. Journal of Environmental Science and Health. Part. B.

[CR25] Kumar M, Yusuf MA, Chauhan PS, Nigam M, Kumar M (2017). *Pseudomonas putida* and *Bacillus amyloliquefaciens* alleviates the adverse effect of pesticides and poise soil enzymes activities in chickpea (*Cicer arietinum* L.) rhizosphere. Tropical Plant Research.

[CR26] Lombard N, Prestat E, van Elsas JD, Simonet P (2011). Soil-specific limitations foraccess and analysis of soil microbial communities by metagenomics. FEMS Microbial Ecology.

[CR27] Lupwayi NZ, Grant CA, Soon YK, Clayton GW, Bittman S, Malhi SS (2010). Soil microbial community response to controlled-release urea fertilizer under zero tillage and conventional tillage. Applied Soil Ecology.

[CR28] Mahapatra B, Adak T, Patil NKB, Pandi GP, Gowda B, Jambhulkar NN, Yadav MK, Panneerselvam P, Kumar U, Munda S, Jena M (2017). Imidacloprid application changes microbial dynamics and enzymes in rice soil. Ecotoxicology and Environmental Safety.

[CR29] Mohiuddin M, Mohammed MK (2013). Influence of fungicide (carbendazim) and herbicides (2, 4-D and Metribuzin) on non-target beneficial soil microorganisms of rhizospheric soil of tomato crop. Journal of Environmental Science, Toxicology and Food Technology.

[CR30] Moreno JL, Aliaga A, Navarro S, Hernandez T, Garcia C (2007). Effects of atrazine on microbial activity in semiarid soil. Applied Soil Ecology.

[CR31] Muñoz-Leoz B, Ruiz-Romera E, Antigüedad I, Garbisu C (2011). Tebuconazole application decreases soil microbial biomass and activity. Soil Biology and Biochemistry.

[CR32] Muñoz-Leoz B, Garbisu C, Charcosset JY, Sánchez-Pérez JM, Antigüedad I, Ruiz Romera E (2013). Non-target effects of three formulated pesticides on microbially-mediated processes in a clay-loam soil. Science of the Total Environment.

[CR33] Niewiadomska A, Skrzypczak G, Sobiech Ł, Wolna-Maruwka A, Borowiak K, Budka A (2018). The effect of diflufenican and its mixture with s-metolachlor and metribuzin on nitrogenase and microbial activity of soil under yellow lupine (*Lupinus luteus* L.). The Journal of Agricultural Science.

[CR34] Orwin K.H., Wardle D.A. (2004). New indices for quantifying the resistance and resilience of soil biota to exogenous disturbances. Soil Biology and Biochemistry.

[CR35] Rahmansyah M, Antonius S, Sulistinah N (2009). Phosphatase and urease instability caused by pesticides present in soil improved by grounded rice straw. Journal of Agricultural and Biological Science.

[CR36] Raju MN, Venkateswarlu K (2013). Impact of pesticides combination on soil microorganisms. Journal of Microbiology and Biotechnology.

[CR37] Saha A, Bhaduri D, Pipariya AS, Basak B (2015). Behaviour of pendimethalin and oxyfluorfen in peanut field soil: Effects on soil biological and biochemical activities. Chemistry and Ecology.

[CR38] Saha A, Pipariya A, Bhaduri D (2016). Enzymatic activities and microbial biomass in peanut field soil as affected by the foliar application of tebuconazole. Environmental Earth Sciences.

[CR39] Sarathchandra SU, Burch G, Cox NR (1997). Growth patterns of bacterial communites in the rhizoplane and rhizosphere of with clover (*Trifolium Repens* L.) and perennial ryegrass (*Lolium Perenne* L.) in long-term pasture. Applied Soil Ecology.

[CR40] Statsoft, Inc., Statistica. (2015) (data analysis software system), version 12.0, www.statsoft.com.

[CR41] Stenrød M, Klemsdal SS, Norli HR, Eklo O (2013). Effects of picoxystrobin and 4-n-nonylphenol on soil microbial community structure and respiration activity activity. PLoS One.

[CR42] Sukul P, Zühlke S, Lamshöft M, Rosales-Conrado N, Spiteller M (2010). Dissipation and metabolism of (14)C-spiroxamine in soil under laboratory condition. Environmental Pollution.

[CR43] Sułowicz S, Piotrowska-Seget Z (2016). Response of microbial communities from an apple orchard and grassland soils to the first-time application of the fungicide tetraconazole. Ecotoxicology and Environmental Safety.

[CR44] Tao L, Yang H (2011). Fluroxypyr biodegradation in soils by multiple factors. Environmental Monitoring and Assessment.

[CR45] Tejada Manuel (2009). Evolution of soil biological properties after addition of glyphosate, diflufenican and glyphosate+diflufenican herbicides. Chemosphere.

[CR46] Tomkiel M, Baćmaga M, Wyszkowska J, Kucharski J, Borowik A (2015). The effect of carfentrazone-ethyl on soil microorganisms and soil enzymes activity. Archives and Environmental Protection.

[CR47] Wang Caixia, Wang Feifei, Zhang Qingming, Liang Wenxing (2016). Individual and combined effects of tebuconazole and carbendazim on soil microbial activity. European Journal of Soil Biology.

[CR48] Wu M, Liu J, Li W, Liu M, Jiang C, Li Z (2017). Temporal dynamics of the compositions and activities of soil microbial communities post-application of the insecticide chlorantraniliprole in paddy soils. Ecotoxicology and Environmental Safety.

[CR49] Wyszkowska J, Tomkiel M, Baćmaga M, Borowik A, Kucharski J (2016). Response of microorganisms and enzymes to soil contamination with a mixture of pethoxamid terbuthylazine. Environmental Earth Sciences.

[CR50] Xu J, Zhang Y, Dong F, Liu X, Wu X, Zheng Y (2014). Effects of repeated applications of chlorimuron-ethyl on the soil microbial biomass, activity and microbial community in the greenhouse. Bulletin of Environmental Contamination and Toxicology.

[CR51] Yang C, Wang M, Cai W, Li J (2012). Bensulfuron-methyl biodegradation and microbial parameters in a riparian soil as affected by simulated saltwater incursion. Clean: Soil, Air, Water.

[CR52] Yuan Xiangyang, Guo Pingyi, Qi Xiang, Ning Na, Wang Hong, Wang Hongfu, Wang Xin, Yang Yanjun (2013). Safety of herbicide Sigma Broad on Radix Isatidis (Isatis indigotica Fort.) seedlings and their photosynthetic physiological responses. Pesticide Biochemistry and Physiology.

